# The role of social status and testosterone in human conspicuous consumption

**DOI:** 10.1038/s41598-017-12260-3

**Published:** 2017-09-18

**Authors:** Yin Wu, Christoph Eisenegger, Niro Sivanathan, Molly J. Crockett, Luke Clark

**Affiliations:** 1 Behavioural and Clinical Neuroscience Institute, Department of Psychology, University of Cambridge, Cambridge, UK; 2 Research Centre for Brain Function and Psychological Science, Shenzhen University, Shenzhen, China; 3 Neuropsychopharmacology and Biopsychology Unit, Faculty of Psychology, University of Vienna, Vienna, Austria; 4 Organisational Behaviour, London Business School, London, UK; 5 Department of Experimental Psychology, University of Oxford, Oxford, UK; 6 Centre for Gambling Research at UBC, Department of Psychology, University of British Columbia, Vancouver, British Columbia Canada

## Abstract

Conspicuous consumption refers to the phenomenon where individuals purchase goods for signalling social status, rather than for its inherent functional value. This study (n = 166 male participants) investigated how the outcome of a social competition influenced conspicuous consumption, and its association with competition-induced testosterone reactivity. Winning a competition increased both explicit and implicit preferences for higher-status vs. lower-status products, using both natural stimuli (prestigious cars) and laboratory-tagged stimuli of matched value (university T-shirts). Competition also influenced behaviour in an Ultimatum Game, such that winners were more likely to reject unfair offers. Competition outcomes had no discernible influence upon salivary testosterone levels, and neither basal testosterone levels nor testosterone reactivity induced by competition predicted the conspicuous consumption measures. Our data indicate that winning a competition lead to more dominant behaviour, albeit in a manner that is not statistically regulated by testosterone, possibly through increased feeling of entitlement.

## Introduction

Why do people purchase luxury goods such as Burberry raincoats or Porsche sports cars? Sometimes, these purchases reflect the inherent value of a high quality product. But a further influence may take the form of status signalling, a phenomenon termed “conspicuous consumption”^1^. Evolutionary psychology claims that conspicuous consumption may be comparable to ostentatious behavioural or morphological traits in non-human animals. Famously, the peacock’s tail is energetically costly to build, but is thought to serve as a reliable and salient signal of genetic quality (c.f. “costly signalling theory”)^2^. By analogy, conspicuous consumption may represent a costly display of wealth that serves to increase an individual’s status within the social hierarchy^3^.

Tentative evidence indicates that testosterone plays a key role in conspicuous consumption. In young men, driving a luxury sport car increased salivary testosterone levels compared to driving an old family sedan^4^. In males, self-reported courtship-related consumption correlated with the second-digit-to-fourth digit (2D:4D) ratio, a putative index of prenatal testosterone exposure, suggesting that men with higher testosterone exposure *in utero* offer more romantic gifts during courtship^5^. Although these studies substantiate the link between testosterone and conspicuous consumption, they leave open the question of whether the effect of testosterone on status-related consumption is causal.

According to the Biosocial Model of Status (BMS)^6,7^, testosterone levels fluctuate as a function of the outcome of competitive interactions. Winning a competition is classically associated with a rise in social status and increase in testosterone levels, whereas losing a competition is associated with a drop in status and testosterone decrease. These outcome-related testosterone changes have important consequences for on-going behaviour: the testosterone increase in winners may serve to promote further dominance behaviours that are required to maintain one’s status. Conversely, testosterone drops among losers may promote submissive behaviours that help to avoid any further loss of status^8^. As evidence for the BMS hypothesis, acute changes in testosterone concentrations within the context of social competition are positively associated with reactive aggression^9^, interpersonal trust^10^ and risk-taking^11^.

The aim of this study was to investigate effects of social status and endogenous testosterone on conspicuous consumption, in a laboratory decision-making context. We used a 2-player Tetris competition that was previously shown to affect testosterone levels^12,13^. Pairs of male participants played against each other in a Tetris contest lasting 15 minutes, and the competitor who scored higher was designated the winner and received a prize. In reality, the outcomes were pre-determined such that participants were randomly assigned to the winning or losing conditions, in order to disambiguate the outcomes from any differences in physical efforts. We hypothesized that testosterone levels would increase among winners and drop among losers.

After playing the Tetris game, participants completed explicit and implicit measures of conspicuous consumption. For the explicit measure, participants provided willingness to pay (WTP) judgments for different products. WTP ratings are used extensively in consumer research, and are sensitive to experimental manipulations^14,15^. For the implicit measure, we used an implicit association test (IAT), an established technique to assess implicit social valuation, that has been used extensively in the study of racial bias and gender stereotypes^16^. In the field of consumer psychology, IAT-measured attitudes can predict brand preferences, usage, and recognition^17^, supporting the validity of IAT in measuring consumption tendencies. Compared to explicit measures, the IAT is less susceptible to social desirability and demand characteristics^16^. Based on the BMS, we predicted that winners would show greater WTP and more positive implicit attitudes for higher-status products compared to losers, and these effects would be mediated by competition-induced testosterone reactivity.

Following our tests of conspicuous consumption, participants played as responders in an ultimatum game (UG). In this classic economic task, two players divide an amount of money between them. The proposer suggests a division of the money, and the responder can either accept or reject the division scheme. If the responder accepts, the money is divided as proposed. If the responder rejects, both players end up empty-handed. Typically, people reject unfair offers (e.g. proposer gets 90% and responder gets 10% of the stake). The purpose of using this task was to demonstrate the effects of social competition by employing an established measure of dominance and aggressive behaviour^18,19^. Previous research has shown that decision-making in the UG is sensitive to status manipulations^20,21^. We hypothesized that winners would reject more unfair offers than losers, as a result of the heightened social status from winning a competition.

## Results

On the Tetris procedure, winners (*M* = 4080.20, *SD* = 3237.10) and losers (*M* = 3891.85, *SD* = 2501.36) did not differ from each other in their true point scores, *t* (160) = 0.41, *p* > 0.25, indicating no systematic difference in expended effort or skill between the two groups. On the PANAS mood ratings, the winners (*M* = 3.90, *SD* = 6.04) showed increased positive affect compared to the losing group (*M* = −2.29, *SD* = 5.45), *t* (160) = 6.84, *p* < 0.001, Cohen’s *d* = 1.08. The winning group (*M* = −1.57, *SD* = 4.52) also showed lower negative affect relative to the losing group (*M* = 0.53, *SD* = 4.74), *t* (160) = −2.88, *p* = 0.004, Cohen’s *d* = 0.45. We looked at the further influence of outcome closeness and basal cortisol levels on the dependent variables (i.e. WTP, IAT, and rejection rate in UG), and there were no significant main effects or interactive terms (see Supplementary Information).

### Competition effect on WTP

We first looked at the effects of competition outcome (winning vs. losing)and product category (cars vs. souvenirs) on self-reported WTP. We used the difference score between WTP for higher-status vs. lower-status products as the dependent variable, with higher (more positive) values representinggreater explicit conspicuous consumption. There was significant main effect of product category, *F* (1, 160) = 10.69, *p* = 0.001, _p_η^2^ = 0.06, such that the WTP difference score was larger for souvenirs (*M* = 1.51, *SD* = 1.72) than for cars (*M* = 0.84, *SD* = 2.35). There was also a significant main effect of competition outcome, *F* (1, 160) = 4.19, *p* = 0.042, _p_η^2^ = 0.03, such that winners (*M* = 1.42, *SD* = 1.69) had a higher WTP difference scorethan losers (M = 0.92, SD = 1.46). The interaction between product category and competition outcome was notsignificant, *F* (1, 160) < 1, p > 0.25.

### Competition effect on implicit attitudes test

We then looked at the effects of competition outcome (winning vs. losing) and product category (cars vs. souvenirs) on implicit attitudes. The scoring algorithm for the IAT used the standard deviations within conditions to compute *D* scores for each experimental condition^22^. Higher *D* scores reflect greater conspicuous consumption by showing facilitated responding when associating high-status products with pleasant words, and low-status products with unpleasant words. There was a significant main effect of product category, *F* (1, 160) = 7.62, *p* = 0.006, _p_η^2^ = 0.05, such that cars (*M* = 0.69, *SD* = 0.40) displayed more positive IAT *D* scores than souvenirs (*M* = 0.59, *SD* = 0.36). There was also a significant main effect of competition outcome, *F* (1, 160) = 5.66, *p* = 0.019, such that winners (*M* = 0.69, *SD* = 0.27) showed more positive IAT *D* scores than losers (*M* = 0.58, *SD* = 0.30), _p_η^2^ = 0.03. Again, the interaction between competition outcome and product category was not significant, *F* (1, 160) < 1, *p* > 0.25.

### Competition effect on rejection rate in UG

In the next step, we investigated the impact of competition outcome (winning vs. losing) and fairness level (£1/£9, £2/£8, £3/£7, £4/£6, £5/£5) on the UG rejection rates. As expected, we observed a reliable effect of fairness level, *F* (4, 640) = 173.98, *p* < 0.001, _p_η^2^ = 0.52, with the rejection rate increasing for more unfair offers. There was a significant main effect of competition outcome, *F* (1, 160) = 4.56, *p* = 0.034, _p_η^2^ = 0.03, with winners (*M* = 38.56%, *SD* = 46.19%) being more likely to reject offers compared to losers (*M* = 29.75%, *SD* = 43.06%). The interaction between competition outcome and fairness level was also significant, *F* (4, 640) = 3.65, *p* = 0.015, _p_η^2^ = 0.03 (see Table 1). Winners were more likely to reject offers in the moderate unfairness range, specifically for £2/£8 offers (*t*(160) = 2.93, *p* = 0.004, Cohen’s *d* = 0.45) and, marginally, for £3/£7 offers (*t*(160) = 1.84, *p* = 0.067, Cohen’s *d* = 0.28).

**Table 1:** Rejection rates for offers at each level [mean (*SD*)].

	£1/£9	£2/£8	£3/£7	£4/£6	£5/£5
Winner	73.80%	67.17%	39.46%	11.14%	1.20%
	(42.21%)	(41.86%)	(45.55%)	(29.28%)	(6.65%)
Loser	63.92%	46.84%	26.90%	9.81%	1.27%
	(45.25%)	(46.58%)	(40.98%)	(26.68%)	(6.82%)

### Association between conspicuous consumption and UG behaviour

The next analysis examined the relationship between the conspicuous consumption measures and the UG rejection behaviour. We used the rejection rate for the £2/£8 offers, where the competition manipulation showed the largest effect. Among winners but not losers, the UG rejection rate was correlated with the WTP difference for cars and the IAT *D* score for cars (see Table 2).

**Table 2:** Correlations between UG rejection behaviour and explicit and implicit measures of conspicuous consumption.

	Cars		Souvenirs	
	WTP difference	IAT *D* score	WTP difference	IAT *D* score
Win	*r* = 0.32	*r* = 0.32	*r* = 0.13	*r* = 0.11
	*p* = 0.003	*p* = 0.004	*p* = 0.243	*p* > 0.25
Loss	*r* = 0.06	*r* = 0.10	*r* = −0.005	*r* = −0.16
	*p* > 0.25	*p* > 0.25	*p* > 0.25	*p* = 0.167

We also looked at the association between the implicit and explicit measures of conspicuous consumption. Collapsing across winners and losers, significant correlations were seen between the WTP difference for cars and IAT *d* score for cars, *r* = 0.26, *p* < 0.001, and between the WTP and IAT d scores for souvenirs, *r* = 0.23, *p* < 0.003, showing conceptual correspondence between the explicit and implicit measures, as would be expected for a trait-like variable reflecting conspicuous consumption. This relationship also held for the winners and losers separately (see Table 3).

**Table 3:** Correlation between the explicit and implicit measures of conspicuous consumption.

	Cars	Souvenirs
Win	*r* = 0.20	*r* = 0.24
	*p* = 0.070	*p* = 0.032
Loss	*r* = 0.30	*r* = 0.19
	*p* = 0.007	*p* = 0.086

### Effects of testosterone

Winning or losing the competition had no observable influence on testosterone fluctuations (see Table 4), *p* = 0.285. Testosterone change and pre-competition testosterone levels were not correlated with any of the four conspicuous consumption indices (see Table 5), all *p*s > 0.1. Given recent studies showing testosterone and cortisol jointly regulate dominant behaviour (“dual hormone hypothesis”)^23^, we also explored interactions between pre-competition testosterone, pre-competition cortisol, and competition outcome (win vs. loss) on conspicuous consumption measurements, all *p*s > 0.1. Neither basal testosterone nor testosterone changes were associated with rejection rate in the UG^24^, all *p*s > 0.1.

**Table 4:** Descriptive statistics for testosterone.

	Winners and losers (n = 156)		Winners (n = 79)		Losers (n = 77)	
	*M* (SEM)	*SD*	*M* (SEM)	*SD*	*M* (SEM)	*SD*
Pre-competition testosterone (pg/mL)	150.31 (3.27)	40.87	146.08 (4.56)	40.54	154.65 (4.67)	41.02
Post-competition testosterone (pg/mL)	150.80 (3.22)	40.18	145.08 (4.36)	38.72	156.67 (4.68)	41.06
Changes in testosterone (pg/mL)^a^	0.49 (2.02)	25.17	−1.00 (2.74)	24.35	2.02 (2.97)	26.06

^a^Post-competition testosterone minus pre-competition testosterone.

**Table 5:** Correlation between testosterone and conspicuous consumption measures.

	Car		Souvenir	
	WTP difference score	IAT *D* score	WTP difference score	IAT *D* score
**Pre-competition testosterone**				
	*r* = 0.08	*r* = −0.05	*r* = −0.03	*r* = 0.106
	*p* = 0.322	*p* = 0.542	*p* = 0.740	*p* = 0.189
**Testosterone changes**				
Winners	*r* = 0.07	*r* = −0.15	*r* = 0.03	*r* = 0.16
	*p* = 0.542	*p* = 0.183	*p* = 0.807	*p* = 0.166
Losers	*r* = 0.11	*r* = 0.06	*r* = −0.05	*r* = 0.09
	*p* = 0.325	*p* = 0.576	*p* = 0.646	*p* = 0.458

### Mediation of mood changes

Given that competition outcomes impacted mood (i.e. PANAS scores) and the cognitive measures, we tested for a mediation effect of mood change. The difference between changes in positive affect and negative affect was used as mediator. The 95% bias-corrected confidence interval for the size of the indirect effect was [−0.5332, 0.0719] for the WTP, [−0.0572, 0.1508] for the IAT, [−0.0768, 0.0966] for rejection rate of £2/£8 offers. As each of these confidence intervals includes zero, we infer no support for a mediating role of mood change between the effect of competition outcome and the cognitive measures.

## Discussion

By using a competition task intended to induce endogenous testosterone fluctuation, this study investigated effects of social competition on conspicuous consumption and bargaining behaviour in the UG, and the potential role of testosterone as an underlying mechanism. Participants who were randomly assigned to be winners of our Tetris competition evidenced both increased self-reported WTP for high-status products, and enhanced implicit bias towards high-status products on the IAT. These explicit and implicit effects were observed for both naturalistic high-status car products (which were also of higher objective value than the lower-status stimuli) but also on our laboratory-tagged status items, which were matched for objective value. A carry-over effect of the competition outcome was also observed on the UG, such that winners were more likely to reject moderately unfair offers. This corroborates previous research showing temporary elevations in social status increased sensitivity to unfairness^20,21^. Despite these robust psychological effects of the competition outcome, our hypothesis that these changes were hormonally regulated was not supported. There was no straightforward winner-loser effect upon testosterone change, although there was evidence of moderation by outcome closeness and basal cortisol levels^25^. Neither basal testosterone nor testosterone reactivity induced by the competition was associated with our conspicuous consumption measures or rejections of offers in the UG.

Social status refers to an individual’s relative position within their social hierarchy in terms of wealth, ability, education, or professional prestige. Competition is a ubiquitous means for determining status^26,27^. In line with the BMS^6–8^, there was a strong competition effect upon subsequent dominance behaviours, manifested by the desire for luxury goods and increased demand for fairness. Certainly, the rejection of unfair offers on the UG is a multiply-determined behaviour, with components including aggression and dominance behaviour, since rejection serves as retaliation against the other player in the face of perceived social provocation (i.e. the unfair offer)^19^. Across winners, conspicuous consumption (for cars) and rejection behaviour were correlated and increased in tandem following the win, implying a shared psychological mechanism underpinned by dominance.

Other research has suggested that conspicuous consumption can serve as a compensatory strategy to restore a sense of lacking power^14^ or under psychological threat^15^. Specifically, individuals experiencing a laboratory induced sense of powerlessness, or self-threat, sought ownership of high-status goods^14,15^. These findings are inconsistent to our observations, in which losing a competition decreased conspicuous consumption tendencies relative to winners. This could be a result of our competition protocol failing to induce a sense of powerlessness or a sense of self-threat, among losers of the competition. Furthermore, in the abovementioned studies, the authors adopted priming procedures in which participants were asked to write a scenario or underwent a short task to elicit the state of powerlessness. Here we employed a 15-minute, intense Tetris competition task, in order engage neuroendocrine systems. The consumption tendencies were measured immediately after priming in the aforementioned studies, whereas we administered the conspicuous consumption tasks 20 minutes after the competition task, given previous research showing saliva testosterone levels peaked around 20 minutes after competition^13^. Future studies are needed to characterize the effect of task features, its impact on related psychological processes, and timing of measures to help reconcile these findings.

The lack of empirical support for testosterone in mediating the effects on conspicuous consumption must be treated with caution, in light of the absence of any discernible difference in testosterone levels on the competition task. In a meta-analytic review of 60 experiments on the “winner-loser effect”, 49% of studies failed to find significant difference in testosterone levels between winners and losers, with some studies even showing a reversed effect^8^. The same paper indicates that laboratory-based studies of competition outcome and testosterone reactivity indicate a relatively small effect, such that winners have elevated T compared to losers (*d* = 0.15). Using G*power software, a sample size of n = 1102 (551 winners, 551 losers) would be required to achieve 80% power to detect significant group differences. Thus, although our sample size was relatively large (*N* = 166) by the standards of the field^12,13^, it was still underpowered. Future studies could directly increase testosterone levels by using testosterone administration^28^, and test its casual effect.

Nevertheless, in the absence of support for a hormonal mechanism, how else might these influences of the competition outcome be generated? One potential mechanism is an enhanced sense of entitlement or deservingness among winners^29^. Psychological entitlement is the feeling that some individuals are more deserving of preferential treatment than others are^30^. A recent study showed that winning a competition lead to dishonest behaviour on a subsequent task, presumably because the increased sense of entitlement induced by winning provided the necessary justification to engage in activities that one would normally consider unacceptable^29^. In a similar vein, our Tetris winners may have felt more deserving of the high-status products and also of fair treatment in the UG. This would be consistent with findings that feelings of “authentic pride” (i.e. superiority over others arising from hard work and success) enhances the desire to purchase luxury brands, as individuals have legitimated their accomplishments and hence felt licensed to purchase luxury goods as a reward^31^. Indeed, an entitlement effect has been proposed to account for increased demand for fairness in asset distribution^32,33^. For example, compared with low status, individuals induced with high status had a higher demand and indicated that they would allocate more to themselves if given the opportunity to act as proposer in UG^20,21^. Future studies are needed to directly measure sense of entitlement or deservedness in the competition task.

It should be noted that WTP was a self-report measure in a hypothetical scenario, thus it has no consequence for the participants. To improve the validity of the conspicuous consumption measures, we measured implicit preference for higher- vs. lower-status products using an IAT, a procedure that is often predictive of consumer behaviour^17^. Importantly, a correlation between WTP and IAT was seen for both naturalist and laboratory-tagged products, highlighting conceptual correspondence between the two measures. We encourage future studies to employ incentive-compatible paradigms in measuring conspicuous consumption, e.g. Becker–DeGroot– Marschak auction^34^.

Social competition is pervasive in our daily life. The present study demonstrated that winning a competition lead to preference for high-status products, possibly through increased feeling of entitlement or deservingness. Characterizing the impact of competition upon decision-making and its associated endocrinological process may therefore enhance our understanding of how competition shapes human social behaviour.

## Methods

### Participants

We recruited 166 male volunteers (mean age = 23.2 years, SD = 3.27, age range = 19–33) from the student population at the University of Cambridge. Sample size was informed by power calculations. Between-group comparisons in groups (winning vs. losing) with 80 participants have a power of 0.85 to detect an effect size of ~ 0.48, which is a plausible effect size based on previous studies on competition-induced testosterone reactivity^35^. The study was conducted in accordance with Declaration of Helsinki and was approved by University of Cambridge Human Biology Research Ethics Committee. Written informed consent was obtained from all participants. We tested male participants exclusively since competition effects are seen to be reliably stronger in males than females^8^. Volunteers attended a single testing session, where they completed the Tetris game (15 min), followed by the conspicuous consumption measures and then the ultimatum game. Volunteers were reimbursed £12 (~US$18) for participation. Results from this dataset on the interactions between competition outcome (win vs. loss), closeness of the outcome (narrow vs. clear) and basal cortisol levels (high vs. low) on testosterone fluctuation were published in a previous paper^25^, but analyses of the carryover effects of competition upon dominance behaviour were not reported (see Supplementary).

### Two-player Tetris Game

The competitive task was adapted from the Tetris game previously used by Zilioli and Watson^12^. Tetris is a speeded puzzle game in which different shapes, dropping down the screen, must be rotated and fitted together into rows. As the game unfolds, the rate at which the blocks drop increases, resulting in steadily increasing difficulty and cognitive effort by the player. Unbeknownst to the participants, the outcome of the task was manipulated such that winning and losing conditions were pre-assigned rather than determined by skill. An important feature of this variant of Tetris was that if the screen completely filled with blocks, the game did not terminate (as in the classic game) but rather the screen would shift the blocks down, allowing all participants to compete for the predetermined amount of time regardless of their prior experience level or ability. After 15 minutes of play, the message “you win!” on a colourful background was displayed on the winner’s screen, while the loser’s screen displayed “you lose!” on a drab background. To intensify the competition, the participants were told immediately before the contest that the Tetris winner would receive an engraved trophy with the text “Tetris winner” and a chocolate bar.

### Validation of the stimulus set

We used two sets of stimuli in the study. Our naturalistic stimulus set was comprised of luxury (higher-status, e.g. Ferrari) and non-luxury cars (lower-status, e.g. Fiat); stimuli common in daily life and familiar to most people. However, a pertinent confound with this manipulation is that the higher-status cars are objectively more expensive than the lower-status cars, and thus socioeconomic variables (e.g. salary) will further influence consumption decisions. For this reason, we developed a laboratory stimulus set of tagged products, in the form of university souvenirs (e.g. T-shirt, mug, hoodie) labelled with university logos from more prestigious North American Ivy-league universities (e.g. Harvard) and less-prestigious state universities; critically, the souvenirs themselves were of equivalent nominal value. Note that we didn’t use British university souvenirs since our participants were from a prestigious British university, and thus potentially confounding their responses.

We validated the experimental stimuli for the conspicuous consumption tasks in an independent student sample (*N* = 89; 34 males). These participants rated the prestige associated with the cars and the university souvenirs (1 = lowest, 9 = highest). In line with our prediction, higher-status cars (*M* = 7.97, *SD* = 0.70) were rated as more prestigious than lower-status cars (*M* = 3.32, *SD* = 1.03), *t* (88) = 33.87, *p* < 0.001 (see Table S1 in Supplemental Material). Similarly, higher-status university souvenirs (*M* = 6.86, *SD* = 1.62) were rated as more prestigious than lower-status ones (*M* = 2.52, *SD* = 0.95), *t* (88) = 36.00, *p* < 0.001.

### Willingness to pay task

Participants were asked to rate, “How much would you be willing to pay for the product featured?”, with 1 = 10% of the retail price of the item, 2 = 20% of the retail price of the item, and increasing intervals of 10% per scale point up to 12 = 120% of the retail price^14^.

### Implicit Association Test

The IAT followed the procedure designed by Greenwald *et al*.^16^, involving two target categories (higher-status vs. lower-status products) and two attribute categories (positive vs. negative). Target categories included higher- and lower-social status products pretested before. Positive attributive words included *beautiful*, *glorious*, *joyful*, *lovely*, *marvellous*, *pleasure*, *superb*, and *wonderful*; negative attributive words included *agony*, *awful*, *horrible*, *humiliate*, *nasty*, *painful*, *terrible*, and *tragic*.

The IAT consisted of seven classification blocks (see Table S2 in the Supplemental Material). In the attribute discrimination task (Block 1, 20 trials), participants were asked to press a left key when a positive word appeared on the screen and a right key for a negative word. Similarly, in the initial target-category discrimination task (Block 2, 20 trials), participants discriminated between higher-status products (responding by pressing left key) and lower-status products (responding by pressing right key). In the initial association task (Block 3, 20 trials practice; Block 4, 40 trials data collection), the attribute and target discrimination requirements were combined, so that participants pressed the left key when either a positive word or a higher-status product was presented, and the right key when a negative word or a lower-status product was presented. In the reversed target-stimuli discrimination task (Block 5, 20 trials), Block 2 was repeated with a switch of the categorization keys by pressing left key when a lower-status product appeared on the screen and a right key when a higher-status product appeared. The reversed association task (Block 6, 20 trials practice; Block 7, 40 trials data collection) again combined two requirements, pressing the left key when either a positive word or a lower-status product was presented and press the left key when a negative word or a higher-status product was presented. Each block started with a brief instruction for the following task and a request to respond as fast as possible while trying to minimize mistakes. Participants were reminded that their error rate and response time would be recorded. The trial sequence was randomized for each participant.

Half of the participants did the seven blocks in the order presented previously; to control for the order effect, Blocks 2, 3, and 4 were swapped with Blocks 5, 6, and 7 for the other half of the participants.

### Ultimatum Game

Participants played 20 one-shot UG via computer interface^36^. To enhance the credibility of the UG task, participants were informed that they were part of a large on-going study in which they would be playing the role of responder with volunteers who had submitted their offers previously. Participants were told that they would receive the financial outcomes from one trial that would be randomly selected at the end of the game. During each trial, participants viewed sequentially a photograph of the proposer (1,000 ms), and the amount of the offer (until response). Participants responded to each offer by pressing one of two buttons (labelled “accept” and “reject”) while the offer was on the screen. There were 4 trials for each of the offer levels (£1/£9, £2/£8, £3/£7, £4/£6, £5/£5 – in this notation, the number before the slash denotes the amount offered to the participant and the number after the slash denoted the amount allocated to the proposer). Unbeknownst to the participants, all the offers were predetermined by a computer program. One trial was randomly selected, and we paid participants on that trial depending on if they chose to accept or reject the offer.

### General procedure

Upon arriving, each pair of participants were greeted by a male experimenter, and each participant was lead to one of two side-by-side testing rooms, where they completed a consent form, a demographic questionnaire, and the Positive and Negative Affect Schedule (PANAS)^37^ to assess mood states prior to the Tetris game. Participants were then instructed to begin the game on the experimenter’s instruction, after which doors to the two testing rooms were shut for the duration of the competition task. The two testing rooms were well soundproofed, so that participants were not aware of the progress of the opponent in the other room. After competing for 15 min, the game’s scripted outcomes were reached and the on-screen feedback was displayed. Following the competition, participants completed the PANAS again, and the WTP, IAT and UG tasks described above. Participants were then debriefed as to the rigged nature of the outcome and were paid the reimbursement.

### Saliva samples and hormone assays

To reduce diurnal hormone variability, all testing occurred between 13:00 h and 19:00 h^38^. After completing informed consent, a demographic questionnaire and PANAS, participants provided a baseline saliva sample (t_0_). They started Tetris competition five minutes (t_5_) after the collection of the t_0_ saliva sample. After completing the competition and revealing the winner and loser (t_20_), participants viewed a neutral video clip (a documentary about Ireland, serving as filler task) in their own test rooms while doors being closed again. At exactly 20 min after the finishing of the Tetris competition (t_40_), participants provided a second saliva sample and then completed the decision-making tasks. Participants were then debriefed as to the rigged nature of the outcome and were paid the participant fees.

Participants were instructed to abstain from eating, drinking, smoking, or brushing their teeth for 1 hour before testing. Saliva samples were collected using passive drool (Salimetrics, Suffolk, England). Samples were chilled immediately following collection, and then frozen within 1 h and held at −80° until assay. Samples were assayed in duplicate using competitive enzyme immunoassays (Salimetrics, Suffolk, England). The average intra-assay coefficient of variation for testosterone was 2.77% (from actual concentrations). The inter-assay coefficients for testosterone averaged across high and low controls were 6.00%. We manipulated the closeness of scores between the two players^25^, and it did not affect any of the dependent variables that we were interested in this study, thus this factor was dropped for the sake of simplicity.

### Statistical analysis

Two participants had extreme low scores on the Tetris task (scoring less than 200 points) and were excluded. One participant reported having major depression and was taking medication, and one participant was found to have participated in the pilot study. Analysis excluded these four participants, leaving 162 participants for the behavioural analysis (83 winners and 79 losers). Inclusion of these four participants did not change the pattern of results. Bonferroni correction was applied to multiple comparisons.

For the hormone data, one participant had blood contamination in the saliva sample, one participant did not provide sufficient saliva sample, and four participants had hormone data differed by more than three standard deviations from the normalized means. These six participants were excluded, leaving 156 participants (79 winners and 77 losers) for data analysis. Baseline (T0) and post-competition (T1) concentrations were normally distributed. Testosterone unstandardized residuals, obtained by regressing post-competition testosterone (T1) against baseline testosterone (T0), were used as measures of testosterone change, as described in Zilioli and Watson^12^.

## Electronic supplementary material

Supplementary Information
