# Supplementary material for: The role of social status and testosterone in human conspicuous consumption
Source: Sci Rep. 2017 Sep 18;7:11803. doi: 10.1038/s41598-017-12260-3 (PMC5603597; doi:10.1038/s41598-017-12260-3)
Supplement: Supplementary file 1 — Supplementary Information [file 41598_2017_12260_MOESM1_ESM.pdf]

The role of social status and testosterone in human conspicuous consumption

YIN WU [1, 2], CHRISTOPH EISENEGGER [3], NIRO SIVANATHAN [4], MOLLY J. CROCKETT [5], LUKE CLARK [6]

[1] Behavioural and Clinical Neuroscience Institute, Department of Psychology, University of Cambridge, Cambridge, UK

[2] Research Centre for Brain Function and Psychological Science, Shenzhen University, Shenzhen, China

[3] Neuropsychopharmacology and Biopsychology Unit, Faculty of Psychology, University of Vienna, Vienna, Austria

[4] Organisational Behaviour, London Business School, London, UK

[5] Department of Experimental Psychology, University of Oxford, Oxford, UK

[6] Centre for Gambling Research at UBC, Department of Psychology, University of British Columbia, Vancouver, British Columbia, Canada

Address correspondence to: Dr. Yin Wu, Research Centre for Brain Function and Psychological Science, Shenzhen University, Shenzhen, China. E-mail: yinwu0407@gmail.com

In accordance with Wu et al. (2017), we analyzed the interactive effects of competition outcome (win vs. loss), outcome closeness (clear vs. narrow), basal cortisol (high vs. low), and product category (cars vs. souvenirs) on the dependent measures.

For the WTP, there was a significant main effect of product category, $F\ (1, 148) = 11.23$, $p = .001$, $_p\eta^2 = 0.071$. The main effect of competition outcome was marginally significant, $F\ (1, 148) = 2.98$, $p = .087$, $_p\eta^2 = 0.02$. There were no other significant main effects or interaction terms in this model, all *p*s > .1.

For the IAT, there was a significant main effect of product category, $F\ (1, 148) = 6.28$, $p = .013$, $_p\eta^2 = 0.041$. The main effect of competition outcome was significant, $F\ (1, 148) = 6.32$, $p = .013$, $_p\eta^2 = 0.041$. There were no other significant main effects or interaction terms in this model, all *p*s > .1.

For the rejection rate in the UG, there was a significant main effect of fairness level, $F\ (4, 592) = 159.21$, $p < .001$, $_p\eta^2 = 0.518$. The main effect of competition outcome was significant, $F\ (1, 148) = 5.83$, $p = .017$, $_p\eta^2 = 0.038$. The interaction between fairness level and outcome was significant, $F\ (1, 148) = 5.01$, $p = .027$, $_p\eta^2 = 0.033$. There were no other significant main effects or interaction terms in this model, all *p*s > .1.

Table S1. Subjective ratings for the stimuli [mean (*SD*)]

Higher-status Car	BWM	Ferrari	Maserati	Mercedes-Benz	Porsche
	7.49	8.53	7.90	7.66	8.28
	(1.14)	(0.74)	(1.67)	(0.95)	(0.87)
Lower-status Car	Fiat	Hyundai	Kia	Suzuki	Vauxhall
	3.48	3.56	2.85	3.24	3.37
	(1.62)	(1.32)	(1.36)	(1.60)	(1.31)
Higher-status Souvenir	Harvard T-shirt	MIT hoodie	Stanford mug	Yale notebook	
	7.02	6.99	6.75	6.67	
	(1.91)	(1.89)	(1.89)	(1.97)	
Lower-status Souvenir	Maryland T-shirt	Miami Hoodie	Minnesota mug	Oregon notebook	
	2.55	2.57	2.69	2.28	
	(1.13)	(1.20)	(1.48)	(1.37)	

Table S2. A list of the categorization tasks in implicit association test

Block	Task (number of trials)	Corresponding key	
		**Left key (F)**	**Right key (J)**
1	Attribute words reaction (20)	Positive words	Negative words
2	Target stimuli reaction (20)	Higher-status products	Lower-status products
3	Initial association task (20)	Higher-status products/positive words	Lower-status products/negative words
**4**	**Initial association task (40)**	**Higher-status products/positive words**	**Lower-status products/negative words**
5	Reversed target stimuli reaction (20)	Lower-status products	Higher-status products
6	Reversed association task (20)	Lower-status products/positive words	Higher-status products/negative words
7	**Reversed association task (40)**	**Lower-status products/positive words**	**Higher-status products/negative words**
